# Associations between park features and adolescent park use for physical activity

**DOI:** 10.1186/s12966-015-0178-4

**Published:** 2015-02-18

**Authors:** Nicole Edwards, Paula Hooper, Matthew Knuiman, Sarah Foster, Billie Giles-Corti

**Affiliations:** Centre for the Built Environment and Health, School of Sport Science, Exercise and Health and School of Earth and Environment, University of Western Australia, 55 Broadway, Nedlands, 6009 WA Australia; Melbourne School of Population and Global Health, The University of Melbourne, Level 5, 207 Bouverie Street, Victoria, 3010 Australia; School of Population Health, University of Western Australia, 55 Broadway, Nedlands, 6009 Perth Western Australia

**Keywords:** Adolescents, Built environment, GIS, Park quality, Parks, Physical activity

## Abstract

**Background:**

Eighty per cent of adolescents globally do insufficient physical activity. Parks are a popular place for adolescents to be active. However, little is known about which park features are associated with higher levels of park use by adolescents.

**Objectives:**

This study aimed to examine which environmental park features, and combination of features, were correlated with higher levels of park use for physical activity among adolescents. By examining park features in parks used by adolescents for physical activity, this study also aimed to create a park ‘attractiveness’ score predictive of adolescent park use, and to identify factors that might predict use of their closest park.

**Methods:**

Adolescents (n = 1304) living in Geraldton, a large rural centre of Western Australia, completed a survey that measured physical activity behaviour, perceptions of park availability and the main park used for physical activity. All parks in the study area (n = 58) were digitized using a Geographic Information System (GIS) and features audited using the Public Open Space Desktop Auditing Tool (POSDAT).

**Results:**

Only 27% of participants reported using their closest park for physical activity. Park use was associated with seven features: presence of a skate park, walking paths, barbeques, picnic table, public access toilets, lighting around courts and equipment and number of trees >25. When combined to create an overall attractiveness score, every additional ‘attractive’ feature present, resulted in a park being nearly three times more likely to be in the high use category.

**Conclusions:**

To increase park use for physical activity, urban planners and designers should incorporate park features attractive to adolescents.

## Introduction

The health benefits of physical activity for children and adolescents are substantial, yet 80% of adolescents worldwide do not achieve the recommended 60 minutes of daily moderate to vigorous physical activity [1]. One way to increase activity levels among adolescents, is to understand the relationship between physical activity and the environments they use for physical activity. One environment where physical activity often occurs is neighbourhood parks. Research shows a greater availability of parks and recreational facilities is positively associated with levels of adolescents’ physical activity [2-5] however, the evidence for park and neighbourhood attributes independently encouraging physical activity among adolescents is limited and results have been mixed [6].

Much is known about what features attract adults to use parks, however less is known about which features attract adolescents to use particular parks. Park features and surrounding environments are typically examined for associations with outcomes such as physical activity levels and weight status [5,7-11]. To date, a range of environmental features have been found to be associated with adolescent park use such as: availability; proximity [12-14]; park size [15]; adolescents’ perceptions of the environment [16]; quality; and use by friends [16].

Other studies have examined the specific park attributes associated with adolescent health behaviours and outcomes. For example, Potwarka et al. found healthy weight status among children (2–17 years) was associated with proximity to specific park features such as the availability of a park playground [10]. By contrast, in a study of 13–15 year olds, Timperio et al. (2008) found the presence of playgrounds within 800 m of participant’s homes was not associated with physical activity [17], possibly because playgrounds were for younger children. Other park attributes associated with higher levels of park use or physical activity among youth, include the presence of: picnic areas; water features [15]; playing fields; [18] basketball courts [15,19]; and again, playgrounds [5,18].

Exposure to greener neighbourhoods and spaces has been associated with lower BMI among children and youth (3–16 years) [20] and higher levels of MVPA among children 8–14 years [21]. However, whether the ‘greenness’ of a park could potentially influence adolescent park use appears not to have been explored to date.

Evidence suggesting that specific park features may influence youth physical activity [22], combined with the inconsistencies in evidence to date, highlights the need for research to better understand the features of parks that attract young people. In addition to individual park features, it is plausible that a combination of park features might be important when designing parks that appeal to adolescent users. For instance, park attractiveness scores have been developed in previous studies of adults, and were found to be associated with higher levels of walking [23,24]. However, given the differences between adults and adolescents in terms of specific park features associated with park use [22] an adolescent-specific park attractiveness score is needed to better understand features of park use that drive adolescent park use.

To understand adolescent park use, parks need to be audited for their features and quality. Traditionally, park features have been captured using audit tools, such as EAPRS [25], POST, CPAT [26,27] and C-POST [28] that require site visits to the park by auditors. More recently, remote sensing [29] and geographic information systems (GIS) techniques have been applied to generate objective measures of park features [30].

Using remote sensing and GIS allows data traditionally captured by on-the-ground audits to be collected remotely. Furthermore, it enables measurement of park features previously not captured in audit tools such as: a measure of park ‘greenness’ using Normalized Difference Vegetation Index (NDVI); the number and size of tree canopies within or around the park; the orientation of surrounding houses; and categorization of surrounding roads. Studies that use GIS to assess the influence of environmental features, such as parks, on physical activity typically measure distances between participant’s residence and their nearest parks or generate park service areas around parks to represent recreation [2,10,11,31-34]. This method assumes people use their closest park however in reality people may choose to travel to use a more attractive park.

The aim of this current study was to examine which environmental park features, and combination of features, correlated with higher levels of park use for physical activity by adolescents. By examining park features in those parks reported as being used by adolescents, this study also aimed to create an attractiveness score that is predictive of adolescent park use for physical activity and identify factors that might predict use of the closest park to where adolescents live.

## Methods

The study was conducted in Geraldton, a large rural centre with a population of approximately 39,000 people, on the coast of Western Australia. It is located 420 km north of the capital Perth.

### Participant data on parks used

Cross sectional data of physical activity attitudes and behaviours were collected in 2006 from 12–15 year olds (*n =* 1304) as part of the ‘*Up4It’* study. The data collection methods and demographic details are described elsewhere [8]. In brief, the Up4It study surveyed all 12–15 year old adolescents living in the Geraldton study area in attendance at one of the five high schools (n = 1626). This larger survey achieved a response rate of 92%, however this paper is based on participants for whom a residential address could be spatially geocoded (n = 1304, 80%). Socio-economic status (i.e., values ranked in deciles) was attributed to each participant according to the Socio-Economic Indexes for Areas (SEIFA) index of Relative Socio-Economic Disadvantage [35]. The scores attributed to each participant were based on the census collection district in which they lived.

Additionally, each participant reported if they were Aboriginal or Torres Strait Islander.

Survey items asked participants: 1) whether they ‘used any park within the last 12 months for physical activity’; and 2) to identify the park they used most often for physical activity.

The number of participants who reported using each park was used as a measure of park use or popularity for physical activity. These data were then used to create a dependent variable representing ‘high’ and ‘low’ use. The top quartile (the 15 parks reported as being used by at least 10 participants) was categorized as ‘high use ‘and the remainder as ‘low use’. The ‘low use’ category included 28 parks that were not reported as being used by any study participant.

### Park layer/audit and access

Using high resolution ortho-imagery of Geraldton sourced from Landgate (flown in 2006 to correspond with the year the survey data collection), all parks within in the study area were manually digitized (*n =* 58) and their size (i.e., area) computed in ArcMap (ESRI v10.1). “Parks” were defined as areas of public open space typically designed for, or able to cater for, a range of different leisure or recreational activities – both active and passive. These included landscaped, ornamental and manicured gardens or parks, as well as publicly accessible (i.e. free to use) recreation spaces, playing fields, ovals and sports surfaces, such as skate parks. Playing fields or sports courts and facilities with restricted access to the public or where the public were not permitted except on payment (i.e. sports clubs or leisure centres or fenced off school grounds) were not included. These were identified using aerial imagery and verified from the sport and recreation listings held by the local government.

All 58 parks identified were audited to assess their features using a modified version of the Public Open Space Desktop Auditing Tool POSDAT [30]. POSDAT captures park attributes using a combination of web-based information and remote sensing methods. This audit tool has been shown to be a reliable and valid desktop method for auditing parks [30]. Additional GIS methods were used to capture the location of playgrounds, the number of trees; the area of tree canopy within the park; the greenness of the park; the proportion of the park perimeters surrounded by lots fronting the park and surrounded by adjacent road types, park size, proximity of the park to the beach and the number of participants who live within a 800 m buffer surrounding the park. Items from POSDAT that have been modified or added to this study are outlined in Table 1. The POSDAT item, *number of trees present,* was previously shown to be valid but had poor inter-rater reliability when tree counts were placed into four categories [30]. To improve the reliability of this item, the number of categories was reduced from four to two, i.e., more or less than 25 trees.

**Table 1:** **Additional POSTDAT items and derived methods**

**Additional POSDAT item**	**Method**
**Activities**	
Any activity space present	A binary variable indicating the presence of any activity space (Yes/No) (from a list of 12 different sports or active recreation spaces captured on PSDAT) was computed. Playground equipment was excluded.
**Environmental quality**	
Number of trees present in park *(Replaces POSDAT item: Estimate the approximate number of trees present 0, 1–50 or 50–100)*	All tree canopies within the parks were manually digitised in ArcMap and the number of trees within each park calculated. For the purpose of this study, the number of trees were placed into two categories <25 trees or >25 trees.
Tree canopy area	All digitised tree canopies within each park were merged in ArcMap to determine a total tree canopy area for each park.
The proportion of the park area covered by tree canopy	The total area of tree canopy was calculated as a proportion of total park area (m^2^). *Total tree canopy area÷park size.*
Path shade	Original POSDAT categories (no pathshade, very poor, poor, medium, good, very good) were collapsed into two categories: High (medium, good and very good) and low (no shade, poor and very poor).
**Amenities**	
Amenity count	The number of different amenities present (includes; barbeques, seating, picnic tables, toilets, public art, lighting) were summed to give a total count per park.
**Safety**	
Park surrounded by minor roads only *Replaces POSDAT item ‘Are all roads surrounding the POS minor roads or cul-de-sacs?’*	Road types were objectively categorised (major or minor) using a classification of major and minor roads according to Western Australian Planning Commission policy and local municipality classification.
	All roads were attributed as major or minor. All roads surrounding each park were identified Parks were coded as being surrounded by minor roads only (Yes/No).
Number of lots surrounding park	All residential lots (cadastre obtained from Landgate, dated 2006) surrounding the parks (i.e., within a 25 m buffer of the park perimeter) were identified and a count of lots surrounding each park computed. [36].
Number of lots orientation ratio	Each of the selected residential lots were inspected and classified according to the orientation of the dwelling on the lot to the park it surrounded; whether it was orientated towards (i.e. facing/fronting) or away from (i.e. backing onto) the park. [36]. The number of residential lots facing the park was divided by the total number of residential lots surrounding the park. Higher ratios (towards 1.0) indicated a higher proportion of the bordering houses being orientated towards the park [36].
Perimeter orientation ratio	The lot orientation ratio was further refined to determine the proportion of the park perimeter that was bordered by lots fronting the park. The length of the park perimeter surveilled by facing cadastre was identified. This was divided by the total perimeter of the park [36].
**Environmental quality**	
Greenness	The presence of greenness in each park was calculated using the Extract Normalized Difference Vegetation Index (NDVI) tool and Landsat TM remote sensing imagery (summer 2006). NDVI provides an indication of the presence and condition of green vegetation with values ranging from −1 to 1. Values of −1 generally represent water, while values of zero correspond to bare surfaces such as rock, sand and roads. Higher values (0.2 to 0.4) represent grassland or bushland. Mean NDVI values for each park were determined.
**Additional items**	
Park size (m^2^)	Using the calculate geometry function in ArcMap the area of each park was determined.
Proximity to beach	Distance calculated between the closest point on park perimeter and closest beach access points manually digitized at sites of beach entry. Distances were calculated using the road/pedestrian network.
No. of participants in 800 m buffer	Points were generated at 10 m intervals around each park and a service area generated extending 800 m from each from each point along the road/pedestrian network . The service area around each of the points was dissolved and the number of participant residential points in the service area was determined.
Giles-Corti adult attractiveness score	The attractiveness score previously used by Giles-Corti et al. (ref) was applied to each park.

Imagery used in this study was flown in summer months to provide the most temporally relevant to the Up4IT survey data collected. Greenness was measured using NDVI (Landsat TM remote sensing imagery captured in summer 2006 and fully calibrated). NDVI provides an indication of the presence and condition of green vegetation with values ranging from −1 to 1. Values of −1 generally represent water, while values of zero correspond to bare surfaces such as rock, sand and roads. Higher values (0.2 to 0.4) represent grassland or bushland.

Mean NDVI values for each park were recoded to tertiles based on distribution of data. NDVI values for parks in this study will be low in general because the imagery is collected during summer. As a result, high values indicate extensive irrigation. Scores in the top third were categorized as high NDVI and scores in the bottom two thirds were categorized as low NDVI.

A pedestrian network was created for all accessibility analyses and is detailed elsewhere [8]. Briefly, the Geraldton street network was edited to include paths and potential shortcuts. Distances between a participant’s residence (measured as the closest intersection in order to comply with the University of Western Australia Human Research Ethics Committee requirements) and: 1) their closest park and 2) the park they reported using most often for physical activity, were determined using network analyses along the pedestrian street network.

### Development of a park quality attractiveness score for adolescents

The associations between ‘high’ and ‘low’ park use for physical activity and park features were examined. Objectively measured park features were individually assessed using crosstabs for categorical variables (with Fisher’s exact test) and ANOVAs for continuous variables. Features not present in the park sample (for example, free tennis courts and wetlands) were excluded from analyses. Features that held stronger associations (*p* value <0.2) with high/low park use for physical activity progressed to the next phase of analysis.

Each park feature was then examined separately for its association with high/low use for physical activity by adolescents in a logistic regression model that included adjustment for park size and the number of participants who lived within 800 m of the park. The seven park features that were significantly and positively associated (*p ≤* 0.05) with park use for physical activity were used to define an attractiveness score relevant to adolescent park use for each park as the number of these seven features that were present in the park. A second park attractiveness score using the combination of features previously established as relevant for adults [23] was also calculated for each park.

### Statistical analyses

Descriptive statistics are presented as percentages for categorical variables and mean (SD) for quantitative variables.

Our measure of high park use was influenced by the spatial distribution of the parks and the participants. As the spatial distribution of parks and participants was not even, we adjusted for number of participants living within 800 m of park when ascertaining the association with park attributes. Additionally, we adjusted for park size to assess the association with park attributes independent of size. However, in a sensitivity analysis we found that the results were essentially the same when we did not adjust for park size.

The strength of the association (odds ratio per additional feature) between each park attractiveness score and high/low park use was estimated from a logistic regression model that adjusted for park size and number of participants living within 800 m of the park.

T-tests were conducted to confirm the difference in the mean attractiveness scores of parks that were reported as being used compared with parks not used at all and the difference between parks with high use and low use.

To explore what variables might predict use of closest park, logistic regression analysis was performed with “closest park is the reported park” as the dependent variable. This analysis was restricted to a sub sample of participants who reported using a park for physical activity (*n =* 751). Independent variables included participant demographic characteristics (age, sex, ethnicity and socio-economic status), attractiveness score of the closest park, distance to closest park and the perception of whether or not there were parks or sporting grounds close to where the participant lived. All data analyses were conducted in SPSS version 19.0.

## Results

### Characteristics of study participants and characteristics of study parks

Demographic characteristics of the study participants (*n =* 1304) and environmental characteristics of the parks audited (n = 58) are presented in Table 2. Participants were evenly distributed among school year groups and genders. Thirteen percent of participants identified themselves as Aboriginal or Torres Strait Islander. Seventy eight percent of participants lived within 800 m of a park.

**Table 2:** **Demographic characteristics of study participants and environmental characteristics of study parks [n (%) for categorical variables and mean (SD) for continuous variables]**

**Demographic characteristics of participants (n = 1304)**	**Number (%)**
School year 8	448 (34)
School year 9	469 (36)
School year 10	387 (30)
Male	633 (49)
Aboriginal/Torres Strait Islander	169 (13)
High SES	618 (47.4)
Proximity to park ≤ 800 m	1026 (78.7)
Reported using park Yes	751 (57.6)
**Environmental characteristics of parks (n = 58)**	**Mean (SD)**
Park size m^2^	16,445 (25 396)
Number of participants within 800 m of park	38.8 (24.7)

The mean park size was 16,445 m^2^ and the mean number of participants who lived within an 800 m service area of a park was 38.

### Development of an attractiveness score for park features associated with high park use

Associations between park attributes and high/low park use are reported in Table 3. Ten items were significantly associated with higher park use (p < 0.2) and were further assessed in the logistic regression models that adjusted for park size and number of participants living within 800 of the park (Table 3). Seven park features remained positively associated with high reported park use for physical activity (*p ≤* 0.05). Most notably, the odds of a park being ‘high use’ rather than ‘low use’ was fourteen times higher if there were public access toilets (OR 13.93; *p* = 0.002), nine times higher if BBQs were present (OR 9.24; p = 0.003), six times higher if there was a skate park (OR 6.41; p = 0.01), and lighting around courts and equipment (OR 6.09; p = 0.012), and nearly seven times higher if there were more than 25 trees present within the park (OR 6.72; p = 0.010). High NDVI levels (i.e., park greenness) also appeared to be associated with higher park use, however this did not reach statistical significant (p = 0.054). The number of activity spaces (i.e., sports courts or surfaces such as basketball courts) within the park was negatively associated with high park use (p = 0.002) and this was dropped from subsequent analyses.

**Table 3:** **The association of POSDAT item and reported ‘high/low park use’ by adolescents and odds ratios from logistic regression models assessing associations between park features (measured using POSDAT and GIS) and high/low park use after adjustment for park size and number of participants living within 800 m of park**

**POSDAT item**	**% of low use parks (n = 43) with POSDAT item present or mean (SD)**	**% of high use parks (n = 15) with POSDAT item present or mean (SD)**	**P-value (one-sided) fishers exact or t- test**	**OR**	**p -value**
*Activities*					
Activity space: Soccer	7.0	0.0	0.422		
Activity space: Football	9.3	7.1	0.643		
Activity space: Cricket	11.6	0	0.230		
Activity space: Baseball	4.7	0	0.566		
Activity space: Fitness circuit	0	7.1	0.246		
Activity space: Basketball/netball hoops	9.3	21.4	0.224		
Activity space: Hockey	2.3	0	0.754		
Activity space: Athletics	2.3	0	0.754		
Activity space: Rugby	2.3	0	0.754		
Activity space: Skateboarding/BMX	4.7	28.6	**0.027**	**6.41**	**0.011**
Children’s playground	67.4	85.7	0.164	4.35	0.125
*Environmental quality*					
Park on river or foreshore	14.0	21.4	0.386		
Water feature: Fountain	4.7	0	0.566		
Water feature: Any	4.7	0	0.509		
Other features: Waterbirds	16.3	14.3	0.614		
Other features: Gardens	20.9	21.4	0.627		
No features	65.1	64.3	0.598		
Number of trees > 25	95.3	85.7	**0.004**	**6.72**	**0.010**
Walking paths present	37.2	71.4	**0.027**	**4.64**	**0.021**
Path shade (High)	4.7	7.1	0.600		
Playground shaded	23.3	42.9	0.224		
Grass reticulated	81.4	92.9	0.288		
High NDVI	25.5	53.3	0.051	3.32	0.054
*Dogs*					
Dogs allowed	95.3	100	0.566		
*Amenities*					
Barbeques	14.0	57.1	**0.003**	**9.24**	**0.003**
Seating	34.9	50.0	0.243		
Picnic tables	37.2	71.4	**0.025**	**5.64**	**0.018**
Public access toilets	18.6	64.3	**0.002**	**13.93**	**0.002**
Public art present	7.0	7.1	0.688		
Lighting: Around courts, buildings, equipment	16.3	50.0	**0.017**	**6.09**	**0.012**
*Safety*					
Park surrounded by minor roads only	72.1	78.6	0.461		
Perimeter orientation ratio*	30.94 (25.00)	32.49 (21.26)	0.831		
Number of lots around park*	10.63 (7.00)	12.00 (8.36)	0.537		
Number of lots facing park*	7.05 (5.94)	8.40 (5.34)	0.440		
Number of lots orientation ratio*	0.56 (0.39)	0.59 (0.32)	0.791		
*Additional items*					
Park size m2*	14786.76 (26944.14)	21199.41 (20378.49)	0.585		
Distance to beach (m)*	1426.65 (2798.61)	1370.81 (1282.16)	0.872		
Tree canopy area*	1647.12 (3461.58)	3083.54 (4462.38)	0.215		
Tree canopy coverage (% total park)*	10.80 (7.75)	14.12 (13.98)	0.266		
NDVI *	0.23 (0.09)	0.29 (0.08)	0.039		
Attractiveness score (Adult) *	32.8 (14.56)	45.7 (13.2)	0.005		
Activity space count*	0.48 (1.05)	0.46 (74)	0.153	0.855	0.002

*Continuous independent variable.

Bold OR and p values = the seven park features included in the adolescent attractiveness score for park use.

The adolescent attractiveness score was therefore based on these seven significant features: the presence of a skate park, walking paths, barbeques, picnic table, public access toilets, lighting around courts and equipment and number of trees >25.

### Confirming associations of park attractiveness scores and frequency of reported park use for physical activity

The mean attractiveness score of parks reported to have been used for physical activity (3.2, SD 1.95) was significantly higher (p < 0.001) than the non-reported parks (1.3, SD 1.17). When comparing parks by their level of reported use (or popularity), the mean attractiveness score of high use parks (4.3, SD 1.78) was significantly higher (p < 0.001) than the low use parks (1.6, SD 1.36).

In logistic regression models that adjust for park size and number of participants living within 800 m of park, the odds of a park being in the high use category was 2.9 times higher (p < 0.001) for every one-point increase in the adolescent park attractiveness score (i.e., for every additional feature present). In comparison, the odds of a park being in the high use category was only 1.1 times higher (p = 0.021) for every one-point increase in the adult attractiveness score.

#### Factors that are predictive of use of closest park

A total of 27% of the 751 participants who reported using a park for physical activity reported using their closet park. Univariate analyses found no associations between age, sex, aboriginality and use of the closest park. Additionally, we found no associations between a park being in a higher socioeconomic area and park attractiveness or having more amenities. However, participants in the higher socioeconomic group were 1.5 times more likely to use their closest park. People who agreed with the statement “There are no parks/ovals close to where I live” were 55% less likely to use their closest park (OR 0.45; 95%CI: 0.23-0.85, p = 0.01). Additionally, the adolescent park attractiveness score was associated with use of the closest park. For every attractiveness point increase in the closest park, the odds of using that park increased by 75% (CI 95% 1.59-1.92, p < 0.001).

## Discussion

There is a dearth of research on specific park features associated with park use for physical activity among adolescents. We found that high park use for physical activity by adolescents was associated with the presence of seven park features: lighting around courts and amenities, a skate park, walking paths, BBQs, picnic tables, public access toilets and a high number of trees. Moreover, odds of using a park with these features was 2.9 times higher for every one-point increase in the adolescent park attractiveness score (i.e., for every additional feature present).

This appears to be the first study to develop an adolescent-specific attractiveness score for parks. We also compared the use of a seven feature adolescent specific park-attractiveness score with our adult attractiveness score, which comprised of nine features. Attributes included in the adult attractiveness score that were not included in the adolescent score were: shade along paths; irrigated lawns; sporting facilities; birdlife; quiet surrounding roads; adjacent to ocean to river and the presence of a water feature. The presence of walking paths and lighting around courts and equipment were included in both scores. We found that while the adult attractiveness score was significantly associated with increased odds of high park use (OR = 1.06), the association was greater when the attractiveness score specific to adolescents was used.

Studies to date have mostly associated park features with physical activity outcomes and traditionally these studies assume that park users frequent their closest park or a park within a specific researcher defined buffer [2,10,11,31-34,37,38]. This current study is unique for its target group because it examined features of parks that participants reported as being used for physical activity. Whilst some studies of adolescents have found the presence of courts to be associated with higher levels of physical activity, [5,19], we found a negative association between the number of activity spaces present and park use for physical activity. Indeed, Byrne and Wolch (2009), suggest that while park attributes may shape use, the extent to which they impact potential user groups is largely unknown [39]. It is possible that in this rural centre, parks or playing fields that provide for a larger number of formal sporting spaces, have less appeal for adolescents who frequent the park for informal activities.

Certain park features of the attractiveness score (such as lighting around courts, buildings and equipment, the presence of a skatepark and walking paths that provide space for roller-skating, skateboarding, bicycling, walking and running) provide physical activity opportunities. Conversely, other features (such as barbeques, picnic tables and a high number of trees) may potentially reduce physical activity by encouraging sedentary behaviour. Yet, our study results indicate if these features are present, the park is more likely to be used by adolescents for physical activity. This may be in part, because adolescents who visit a park for physical activity may also require social amenities (such as toilets, picnic tables for seating and trees for shade) to remain in the park for longer periods of time, thus providing opportunity for prolonged participation in physical activity. Moreover, the presence of these amenities may encourage greater use by the community for a wide range of activities: greater use may attract other park users [23].

The use of GIS and remote sensing techniques enabled new park features to be objectively measured, further expanding the items previously collected with POSDAT. For example, the number of participants living within an 800 m buffer of the park, tree canopy coverage and the proportion of the park perimeter that had residential dwellings facing the park were all objectively measured using GIS rather than relying on auditing. Notably, a ‘higher number of trees’ in the park was significantly associated higher park use for physical activity. Whilst the other new items were not significantly associated with high park use in this study, future studies may also wish to include these measures, as associations may be found in studies with a larger sample size, more varied environments and/or with a different target groups (e.g. older adults who may only use public open space nearer to their home).

The greenness of a park may be an important feature that influences an adolescent’s choice of parks. Whilst the calculation of NDVI did not achieve the conventional levels of significance in this study (p = 0.054), this may be due to the small sample size of parks or the lack of variation in the sample. It could also be due to the measurement limitation of using NDVI for small parks. For example, no standard deviation of NDVI could be determined because the pixel size required for NDVI calculation (30 m × 30 m) was larger than some of the parks. Other studies however have found greenness is important therefore future studies may wish to include NDVI in studies of public open space and adolescents.

Examining features of parks actually used for physical activity, as opposed to the park closest to home, is recommended for studies examining the relationship between parks and physical activity. Notably, we found that less than a third of participants used the park closest to where they lived. Indeed, the perception “there are no parks close to where I live” was negatively associated with use of the closest park whereas park attractiveness was positively associated with use of the closest park. Those living in higher SES areas also were more likely to use their closest park. However, we found no association between parks in higher socioeconomic areas and park attractiveness, or having a higher amenity count. This is in contrast to the findings of Crawford and colleagues in Melbourne, Australia who found parks in higher socioeconomic areas are more likely to be more attractive and have more amenities [28].

This study is limited by the small number of parks included in the sample however this was unavoidable, given our study was set in a in a rural location. Furthermore, as a result of being a rural setting some POSDAT items could not be examined because they were not present in any of the parks in the study areas. Future studies undertaken in larger rural centres, should consider using all of the POSTDAT items, rather than those items used in this study. Some caution should be exercised when using orthoimagery to audit parks because the time of the year the imagery is captured may not reflect park conditions all year round. Another limitation of our study is that park use varies by season and associations found in this study were not stratified by season.

This focus of this study was on assessing park access, reported as being used from participants’ residential locations. Further research may take into account the distance from participants’ school or the park’s location in terms of being along routes between home and school locations. Moreover, this study examined environmental characteristics of parks however, as suggested by Byrne and Wolch (2009), cultural preferences of potential park users should also be considered [39]. Finally, although this study found associations between objective park measures and levels of use, it is recommended that future studies also consider using both objective and subjective measures of park quality to better understand what influences park use among adolescents.

## Conclusions

Park use for physical activity by adolescents was associated with seven features: presence of a skate park, walking paths, barbeques, picnic table, public access toilets, lighting around courts and equipment and number of trees >25. These features were combined to create an overall adolescent-attractiveness score, where for every additional feature present, parks were almost three times more likely to be used by adolescents. Further our adolescent attractiveness score was a better predictor of park use for physical activity than a previously developed adult-specific park attractiveness score. Given such a large number of adolescents do not meet recommended guidelines for physical activity, landscape architects might consider designing parks with such features to encourage increased adolescent physical activity through park usage.

## Abbreviations

GIS: Geographic Information System
POSDAT: Public Open Space Desktop Auditing Tool
NDVI: Normalised Difference Vegetation Index
